# SunGold Kiwifruit and Psychological Health (GoKiPH): A Randomised Controlled Crossover Trial

**DOI:** 10.3390/nu17081375

**Published:** 2025-04-18

**Authors:** Michael Billows, Naomi Kakoschke, Ian T. Zajac

**Affiliations:** 1 Human Health, Health and Biosecurity, CSIRO, Adelaide, SA 5000, Australia; naomi.kakoschke@csiro.au (N.K.); drianzajac@gmail.com (I.T.Z.); 2 School of Psychology, University of Adelaide, Adelaide, SA 5005, Australia

**Keywords:** kiwifruit, psychological wellbeing, mood, vitamin C

## Abstract

**Background/Objectives:** The consumption of SunGold kiwifruit, a fruit rich in vitamin C, has been associated with improved mood in healthy individuals with low vitamin C levels. However, no studies have examined this relationship in individuals with elevated mood disturbance. This study examined the potential for SunGold kiwifruit to improve psychological wellbeing in mood-disturbed adults. **Methods:** This study was a two-period, non-blinded crossover trial. Adults (*n* = 26) aged 18–60 years with mild to moderate mood disturbance were randomised with a two-week washout between periods. During each 4-week period, participants consumed either two SunGold kiwifruit daily or their usual diet. The primary outcome was mean change in total mood disturbance scores from the kiwifruit period compared to the diet-as-usual period. Secondary outcomes were blood plasma vitamin C concentration, wellbeing, vitality and gastrointestinal symptoms. Participants and researchers were unblinded to condition and intervention. **Results:** Scores for total mood disturbance (65.2%, *p* < 0.001), wellbeing (10.5%, *p* < 0.01) and vitality (17.3%, *p* = 0.001) significantly improved in the kiwifruit condition compared to the usual diet. Vitamin C (27.5%, *p* = 0.002) concentrations also improved and gastrointestinal symptom reduction was evident during kiwifruit consumption (16.2%, *p* = 0.003). There were no serious adverse events. SunGold kiwifruit consumption resulted in significant reductions in total mood disturbance scores and improvements in wellbeing, vitality and vitamin C concentrations. Gastrointestinal symptom severity also significantly reduced. **Conclusions:** Results provide preliminary evidence of the potential benefits of kiwifruit for reducing mood disturbance in adult populations. Further studies in diverse groups, including clinical populations, are warranted.

## 1. Introduction

Mental health disorders were amongst the top ten leading causes of disease burden worldwide in 2019. The Global Burden of Disease Study 2019 estimated that major depressive disorder and dysthymia jointly contributed to 46.9 million disability-adjusted life years [1]. Depression is one of the most common mental health disorders in the general population [2]. Recent global estimates have reported a 27.6% increase in depression post-COVID-19, such that global prevalence is now 3.2% [3]. Despite the high prevalence rates, approximately 30% of individuals with depression receive treatment and of those, at least 60% do not receive adequate treatment [4]. Hence, there is a pressing need to deliver efficacious, accessible treatments for depression to reduce the individual and global burden. Beyond the treatment offered by specialised mental health services (e.g., psychological or pharmacological treatments), there are a growing number of adjunctive treatments gaining consumer and empirical support [5].

Associations between mental health and diet quality are well established, particularly in relation to fruit and vegetable consumption [6]. Results from cross-sectional studies and clinical trials highlight that diets comprising higher daily servings of fruits and vegetables were positively associated with psychological wellbeing, vitality, flourishing, mood and reduced depressive symptoms [7,8,9]. Raw fruits and vegetables such as dark leafy greens, bananas, apples and kiwifruit, are associated with better mental health outcomes, most likely because they deliver greater amounts of nutrients than when cooked or canned [7]. Clinical trials that have examined the psychological wellbeing-diet dyad at the micro-nutrient level (e.g., vitamin C) also report positive associations between mood and wellbeing [10,11,12].

Vitamin C is one micro-nutrient that has received considerable attention in the diet and wellbeing literature. Vitamin C is a water-soluble vitamin that is not endogenously produced in humans despite being absolutely required for a range of important biochemical functions [13]. Therefore, vitamin C must be derived from exogenous sources, principally through the consumption of fruits and vegetables [14]^.^ Vitamin C is more highly concentrated in the brain compared to plasma, and is thought to play an important biochemical role in psychological wellbeing by acting directly and indirectly in the synthesis of neurotransmitters such as serotonin, dopamine and oxytocin [15]. Vitamin C supplementation has been associated with reduced mood disturbance, psychological distress and fatigue, as well as improved mood and vigour in heterogenous samples [16,17,18]. Conversely, sub-optimal vitamin C status (<50 µmol/L) and clinical deficiency (<10 µmol/L) are associated with fatigue, irritability and low mood [19,20]. As vitamin C is inherently unstable and easily destroyed through processes such as cooking and prolonged storage, the inclusion of vitamin C-rich foods (e.g., certain fruits and vegetables) in habitual diets is recommended to maintain adequate concentrations and maximise vitamin C bioavailability [21].

Kiwifruit is widely acknowledged as a vitamin C-rich fruit, in addition to containing nutritionally relevant levels of vitamin E, dietary fibre, potassium and folate [22]. The two cultivars of greatest current commercial significance are the *Actinidia deliciosa* “Hayward” (Green kiwifruit) and the *Actinidia chinensis* var. *chinensis* ‘*Zesy002*’ (SunGold kiwifruit). The green kiwifruit contains ~88 mg of vitamin C, whilst the SunGold kiwifruit contains ~152 mg of vitamin C per 100 g flesh weight, more than three times the Australian recommended daily vitamin C intake (45 mg) [23]. Compared to other commercially available fruits, the vitamin C content of SunGold kiwifruit eclipses levels found in oranges (52 mg), strawberries (46 mg), pineapple (21 mg), bananas (5 mg) and blueberries (4 mg) per 100 g [24]. In recognition of the substantial vitamin C content of gold kiwifruit, an emerging subset of studies have examined the potential for vitamin C-rich gold kiwifruit to increase vitamin C concentrations and improve psychological health.

Intervention trials have demonstrated that gold kiwifruit consumption increased blood plasma vitamin C concentrations at different dose rates and in samples with adequate (>50 µmol/L) and sub-optimal vitamin C levels (23–50 µmol/L). For example, consuming as little as half a gold kiwifruit (equivalent to ~50 mg vitamin C) daily for six weeks significantly increased blood plasma vitamin C from 23 µmol/L to 46 µmol/L (*p <* 0.001) in young adult males with sub-optimal vitamin C status (*n =* 36) [25]. Comparable increases in vitamin C concentration (38 µmol/L to 62 µmol/L, *p <* 0.05) were observed when young adult males (*n =* 15) with sub-optimal vitamin C status consumed two gold kiwifruit daily for six weeks [26]. Healthy adults aged 44–85 years (*n =* 26) with adequate vitamin C status recorded significantly higher plasma vitamin C concentration after consuming two SunGold kiwifruit daily for twelve weeks [27]. Findings of these studies are supported by extended lead-in periods to stabilise dietary intake and weekly vitamin C analysis [25,26]. Generalisability to other populations (e.g., over 35-year-olds, non-students, adults with mood disturbance) is limited and only one study included a mixed-gender sample [27].

Nascent research has begun to examine the potential benefits of kiwifruit-delivered vitamin C on psychological health and has returned promising results. Carr et al. [28] randomised young adult males with sub-optimal vitamin C status (<50 μmol/L) (*n* = 35) to receive either half a gold kiwifruit or two gold kiwifruit daily for six weeks. Participants who consumed two gold kiwifruit significantly increased vitamin C levels (*p <* 0.001) and displayed a trend towards decreased mood disturbance and reduced symptoms of depression. A sub-group analysis of participants (*n* = 8) with higher-than-average mood disturbance showed significant 38% decreases in mood disturbance and fatigue (*p* = 0.029 and *p* = 0.048, respectively), a 31% increase in vigour (*p* = 0.024) and a 34% trend towards decreased depression, whilst no such effects were observed in those with lower-than-average mood disturbance (*n* = 9) following supplementation with two gold kiwifruit. More recently, Conner et al. [29] conducted a randomised, three-arm clinical trial involving adults aged 18–35 years (*n* = 167) with sub-optimal vitamin C status (<40 μmol/L). Participants consumed either two SunGold kiwifruit, an equivalent-dose vitamin C supplement (250 mg) or a chewable placebo matched for appearance and flavour to the supplement daily for 28 days. Participants who consumed two SunGold kiwifruit had significantly increased vitamin C concentration (*p* < 0.001) and reported reduced mood disturbance (*p* = 0.026) and improved wellbeing (*p* = 0.052), with wellbeing improvements preserved across the two-week washout relative to both of the other groups.

Previous studies [28,29] examining the effect of gold kiwifruit consumption provide preliminary evidence of benefits for vitamin C status and mood disturbance. Findings of the two studies are strengthened by the inclusion of validated psychometric instruments, mechanistic vitamin C assessment and intervention periods of sufficient length to assess indices of change. However, the generalisability of results is limited by the small sample size of young adult males and under-powered sub-analysis [28], restriction of the samples to 18–35 year olds and lack of blinding. Therefore, the generalisability of those results to other populations, such as individuals with elevated mood disturbance, is unclear. The current trial sought to expand on previous findings by examining the influence of gold kiwifruit consumption on vitamin C status and mood in participants with mild to moderate mood disturbance, a cohort who might arguably derive greater benefit than other populations. The principal aim was to examine whether the consumption of SunGold kiwifruit twice daily for 28 days improved psychological wellbeing in mood-disturbed adults. Secondary outcomes included blood plasma vitamin C concentration, wellbeing, vitality and gastrointestinal symptoms.

## 2. Materials and Methods

### 2.1. Trial Design

**Figure 1:**
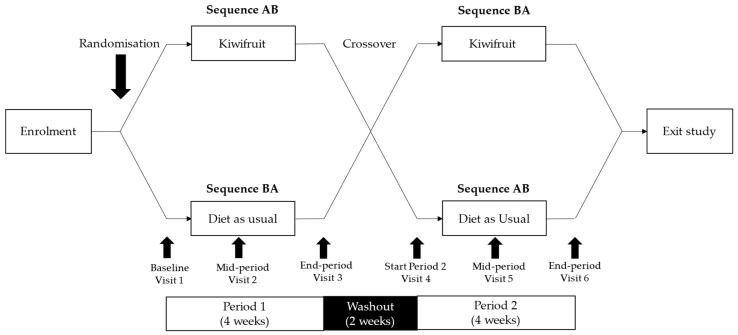
Schematic overview of GoKiPH trial.

The Gold Kiwifruit and Psychological Health (GoKiPH) trial (Australian New Zealand Clinical Trials Registry: ACTRN12622000259741p, approved on 14 February 2022) was a two-period, non-blinded crossover trial with participants randomised to a counter-balanced sequence. Participants consumed 2 SunGold kiwifruit daily or their typical diet for four weeks, with a two-week washout between periods (Figure 1). There were no changes to study protocol after trial commencement.

### 2.2. Participants

Eligibility criteria are detailed in Table 1. Participants aged between 18 and 60 years old with mild to moderate mood disturbance were recruited through the Commonwealth Scientific and Industrial Research Organisation (CSIRO) Research Clinic in Adelaide, Australia, from June to October 2022. The study was promoted on the CSIRO Facebook page and Research Clinic website and advertised for adults experiencing current symptoms of depression and anxiety to submit an application to determine suitability for enrolment.

### 2.3. Procedure

After responding to study advertisements, interested applicants were directed through a two-stage screening process that incorporated a medical screening questionnaire followed by a telephone-delivered assessment of mood (Kessler Psychological Distress Scale, K10) [30].

Participants attended six fortnightly in-clinic visits. At each visit, participants provided a fasting blood sample and completed psychometric and gastrointestinal symptom questionnaires, and basic anthropometric measures were documented. At the first and second visits during the kiwifruit intervention period, participants received a two-week supply of SunGold kiwifruit, along with instructions on the storage, consumption and disposal of unused fruit. They were also provided with a compliance and health log to record consumption and adverse events. Participants were instructed to consume their usual diet during the two-week washout period.

### 2.4. Intervention

Participants consumed two Zespri SunGold kiwifruit (‘Zesy002^TM^’, *Actinidia chinensis* var. *chinensis*) daily for 28 days. Kiwifruit were sourced from Zespri International Ltd, Mt. Maunganui, New Zealand. Participants consumed the kiwifruit at a time of their choosing, either eating both in a single sitting or separately throughout the day (i.e., one at a time). Participants were instructed to store the kiwifruit in domestic refrigeration to maximise ripeness, and to remove the skin prior to eating (consume the flesh only).

### 2.5. Outcome Measures

Profile of Mood States Short Form (POMS-SF)

The POMS-SF [31] is a self-report measure that contains a list of 35 mood-related adjectives. Respondents are asked to indicate the degree to which each item reflected their experience during the preceding seven-day period. Items are rated on a five-point Likert-type scale ranging from 0 (not at all) to 4 (extremely). The mood-related items reflect six factors of tension–anxiety, depression–rejection, anger–hostility, vigour–activity, fatigue–inertia, and confusion–bewilderment. A total mood disturbance (TMD) score is calculated by subtracting the value of the vigour–activity sub-scale from the sum of the remaining five sub-scales (minimum score −20 to maximum score 100), with higher scores indicating greater mood disturbance (Cronbach’s α = 0.87–0.92).

Warwick–Edinburgh Mental Wellbeing Scale (WEMWBS)

The WEMWBS [32] is a 14-item scale that measures subjective wellbeing and psychological functioning in the previous two-week period. Positively worded statements such as ‘feeling good about myself’, ‘thinking clearly’, and ‘feeling relaxed’ are rated on a Likert-type scale ranging from 1 (none of the time) to 5 (all of the time). The 14 items are summed to produce a total wellbeing score (minimum score 14 to maximum score 70) with higher scores reflecting greater levels of wellbeing (Cronbach α = 0.89).

Subjective Vitality Scale (SVS)

The six-item version of the SVS [33] assesses feelings of subjective vitality and includes statements that ask respondents about their feelings of feeling energised, looking forward to each day and feelings of aliveness. Each positively worded statement is rated on a seven-point Likert scale from 1 (not at all true) to 7 (very true) with all items summed to produce a total vitality score (minimum score 6 to maximum score 42), with higher scores reflecting greater vitality (Cronbach α = 0.85).

Gastrointestinal Symptom Rating Scale (GSRS)

The GSRS [34] is a 15-item scale that asks respondents to rate the severity of common gastrointestinal symptoms such as heartburn, bloating and constipation during the preceding seven-day period on a seven-point Likert scale from 1 (no discomfort at all) to 7 (very severe discomfort). The 15 items reflect five domains (abdominal pain, reflux syndrome, diarrhoea syndrome, indigestion syndrome and constipation syndrome) of gastrointestinal symptoms, with higher scores indicating greater symptom severity (Cronbach α = 0.61–0.83).

Blood plasma vitamin C analysis

Participants fasted for a minimum of 10 h prior to providing a sample at in-clinic appointments conducted between 7.30 a.m. and 9.00 a.m. on the morning of their appointment. A 5 mL blood sample was drawn directly into a lithium heparin tube, wrapped in foil to protect the sample from light and placed on ice while awaiting collection. Once collected, samples were frozen and stored at temperatures below −18 °C while awaiting analysis. After thawing, the sample was instantly prepared for analysis, and a determination of vitamin C was performed from lithium-heparin plasma and serum. For analysis, 100 μL of Precipitant P was combined with 100 μL serum and mixed for 30 s on a vortex mixer prior to incubation for 10 min at 4 °C. The sample was subsequently centrifuged for 10 min at 10,000× *g*, and the vitamin C content of a sample (20 μL) of the resulting supernatant was determined by high-performance liquid chromatography using ultra-violet detection [35].

### 2.6. Sample Size

It was determined that a sample size of *N* = 60 participants would be required for a repeated-measures model, assuming a small within-and-between interaction effect (η^2^ = 0.02) with 80% power (α = 0.05). Using a crossover design, each participant serves as their own control, thus providing high power and statistical efficiency while needing half the number of participants necessary for a between-subjects design. Thus, a minimum sample size of *n* = 30 was required to sufficiently power the study. A previous clinical trial that used an identical intervention as well as primary mood outcome measures and vitamin C analysis reported an attrition rate of 7% (equivalent to *n* = 2 in the present trial), albeit in a non-mood-disturbed sample [29]. An additional 10% attrition (*n* = 4) was apportioned to allow for dropouts, given the mood-disturbed population the trial was seeking to recruit, leading to a required sample size of *n* = 36.

### 2.7. Randomisation, Allocation and Blinding

Participants were block-randomised to sequence in a 1:1 allocation using a computer-generated algorithm by a co-author (ITZ). The lead researcher (MB) was responsible for enrolling and assigning participants to sequence. Researchers and participants were unblinded to sequence allocation and the intervention.

### 2.8. Data Analysis and Statistical Methods

Statistical analyses were performed using R version 2023.6.1.524 [36]. In the event of missing data for the psychological (POMS-SF, SVS and WEMWBS) and gastrointestinal (GSRS) questionnaires, these were replaced using the intra-individual scale mean when at least 50% of scale items were completed [37]. Missing data were mainly due to dropout (*n* = 3; *n* = 1 sequence AB, *n* = 2 sequence BA) (see Figure 1), which all occurred by week 2. Missingness on individual scales across the sample (*n* = 26) did not exceed 15% (minimum 2.8% to maximum 14.3%). A mixed effects linear regression model was adopted for primary and secondary outcomes to estimate change in the outcome variable under kiwifruit supplementation and diet-as-usual conditions. Using the LMER function within the LME4 package in R [38], models were specified to include main effects for sequence allocation, treatment, time and a treatment*time interaction. In addition, two random effects specifications were compared to identify the optimal model for the primary outcome (TMD) and included (1) a model with random intercepts for each participant (1|SubjectID) and (2) a model permitting variation in the treatment effect across subject/sequence combinations (Treatment|SubjectID:Sequence). The model permitting differential effects across participant/sequence combinations performed statistically better (chi-square = 11.73, *df* = 2, *p* = 0.002) and was retained for all subsequent models.

## 3. Results

### 3.1. Recruitment

**Figure 2:**
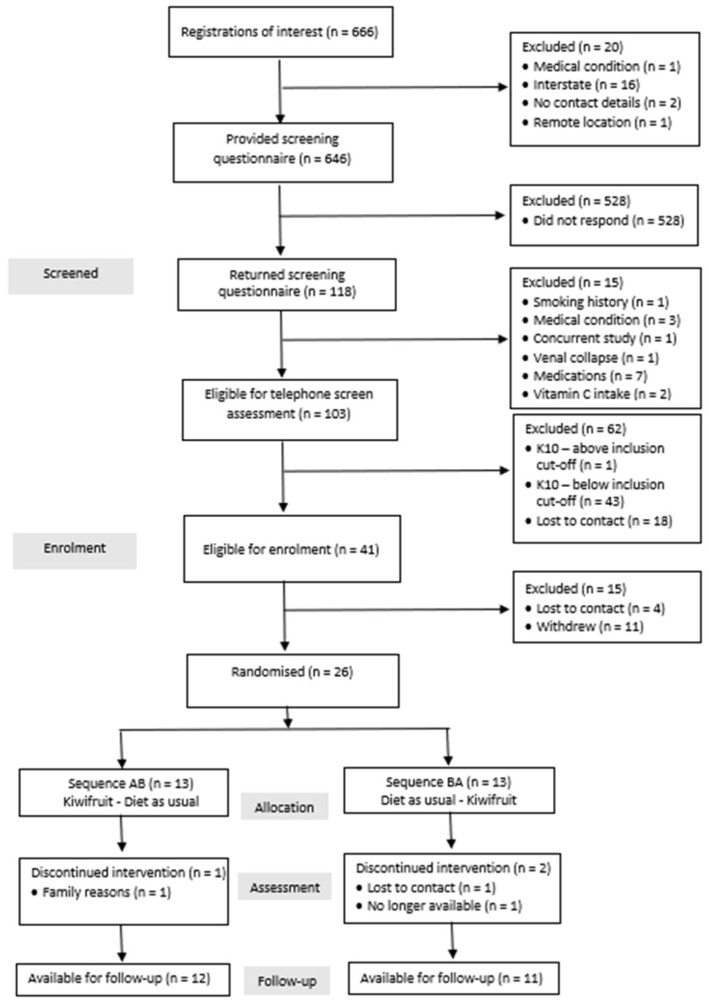
CONSORT flowchart of movement of participants through screening, enrolment, allocation and follow-up.

This study received a considerable amount of interest (Figure 2), and 17.7% (118 of 666) of applicants who expressed an interest in participation returned the medical screening questionnaire. Fifteen applicants (12.7%) were excluded based on medical eligibility criteria. Additionally, 44 (42.7%) of the remaining 103 applicants were excluded following psychological screening and a further 18 (17.5%) were lost to contact. Then, 15 (36.6%) of the remaining 41 applicants either withdrew (*n* = 11) or were lost to contact (*n* = 4), leaving 26 participants eligible for enrolment.

### 3.2. Participant Characteristics

Twenty-six participants were sequentially enrolled in the trial between June and October 2022, with *n* = 13 participants allocated to each sequence. Three participants withdrew from the study. One participant (sequence AB) withdrew after the second visit, citing family health issues, and two participants from sequence BA withdrew after completing the baseline visit (lost to contact *n* = 1 and work commitments *n* = 1). The mean age of the kiwifruit and diet-as-usual conditions were not dissimilar. The sample was predominantly female ~75% and the mean BMI was in the overweight range (25.0–29.9) (Table 2). The two conditions were moderately mood-disturbed on a scale from −20 to 100, with higher scores indicating greater mood disturbance. At baseline, the participants’ blood plasma vitamin C concentrations ranged from 15 µmol/L to 110 µmol/L, with the mean blood plasma concentrations indicating that participants were not vitamin C deficient at baseline. The mean wellbeing scores were moderate, with higher scores indicating greater wellbeing (minimum 14 to maximum 70), as was the mean vitality score, with higher scores reflecting greater vitality on a scale from 6 to 42. Gastrointestinal symptom ratings for each group were considered low on a scale from 15 to 95, with higher scores indicating greater symptom severity.

### 3.3. Adverse Events and Compliance

No serious adverse events were recorded. One participant reported flatulence and mild bloating that persisted for three weeks during the kiwifruit intervention; however, this did not affect participation or compliance. Phlebotomy staff were unable to draw blood from one participant on two occasions, and on a single occasion for a second participant. Compliance with study product consumption was high (*n* = 26, 97.4%) and did not differ between sequences (*p* > 0.05).

### 3.4. Primary Outcome-Total Mood Disturbance

**Figure 3:**
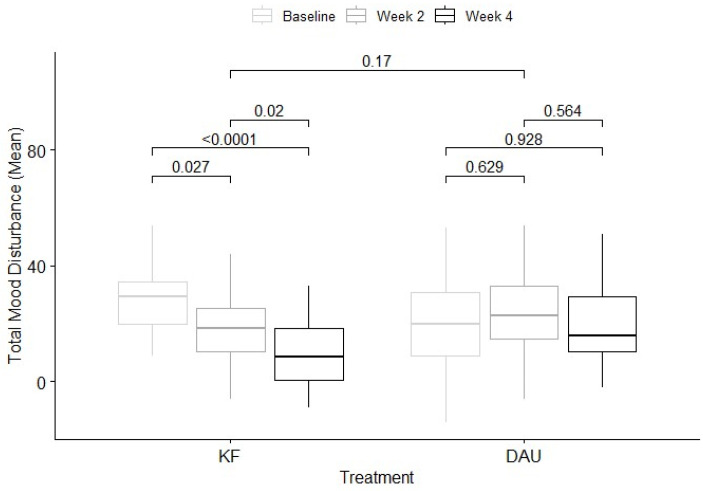
Mean difference (SE bars) in total mood disturbance at week 2 and week 4 for kiwifruit (KF) and diet-as-usual (DAU) conditions.

Linear mixed effects models for primary and secondary outcomes are presented in Table 3. Means and standard deviations are presented at Table 4. The model examining the effect of the intervention on total mood disturbance showed a significant time effect, *F*(2, 112.88) = 7.96, *p* < 0.001, but not a significant overall treatment effect, *F*(1, 114.17) = 3.34, *p* = 0.07. However, the interaction between treatment and time was significant, *F*(2, 112.87) = 9.52, *p* < 0.001 (Figure 3). Mean total mood disturbance scores reduced significantly between baseline (*M* = 27.6, *SD* = 13.5) and week two (*M* = 18.6, *SD* = 13.5) and week four (*M* = 9.6, *SD* = 12.0) in the kiwifruit condition. Scores for the diet-as-usual condition did not change significantly across timepoints.

### 3.5. Secondary Outcomes

**Figure 4:**
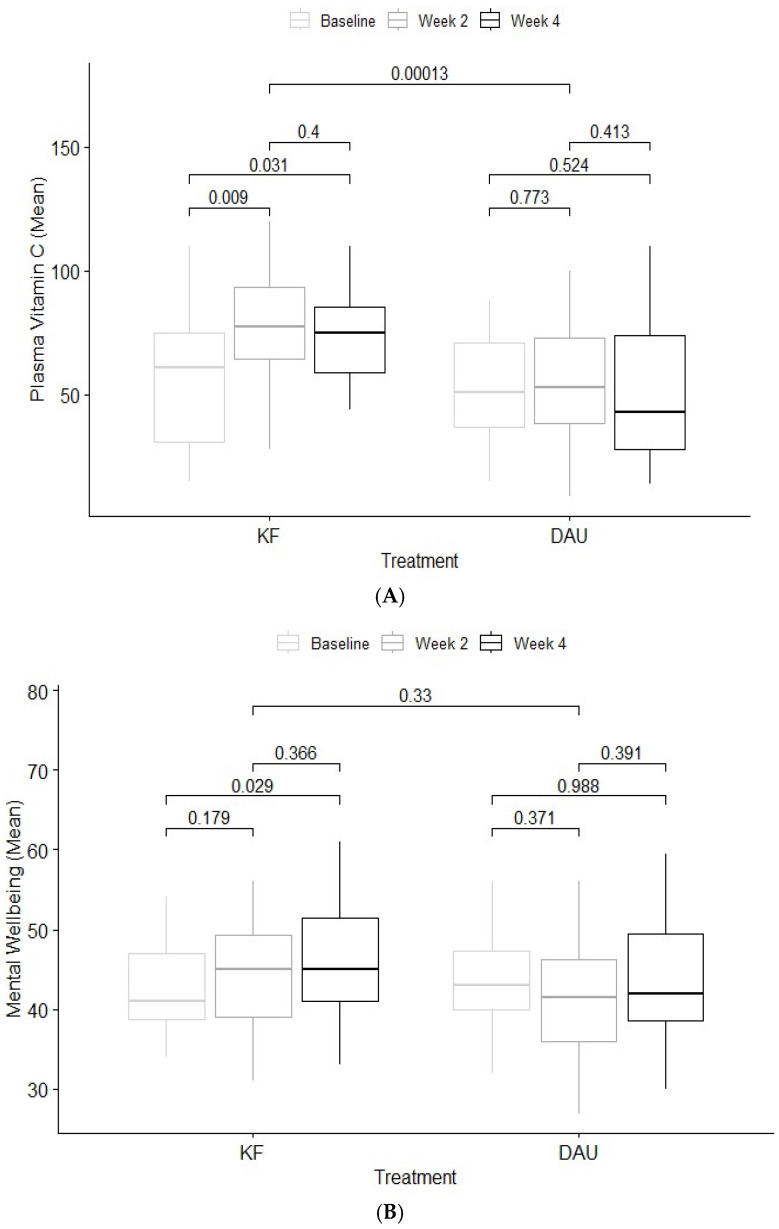

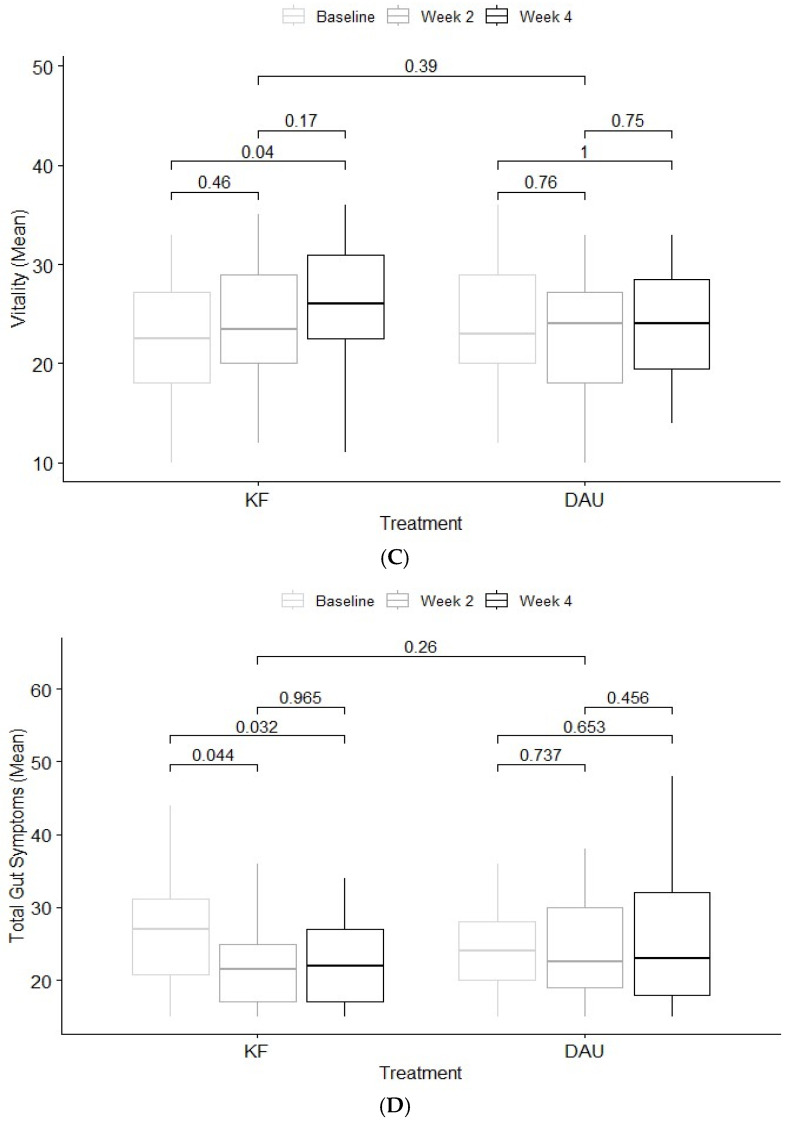
Mean difference (SE bars) in plasma vitamin C (**A**), wellbeing (**B**), vitality (**C**) and gastrointestinal symptoms (**D**) at week 2 and week 4 for kiwifruit (KF) and diet-as-usual (DAU) conditions.

Regarding plasma vitamin C (Figure 4A), results showed a significant time effect, *F*(2, 83.28) = 5.39, *p* = 0.006, and a significant overall treatment effect, *F*(1, 23.03) = 18.92, *p* < 0.001. The interaction between treatment and time was significant, *F*(2, 83.42) = 3.91, *p* = 0.02. Mean vitamin C concentration (µmol/L) increased significantly at week two (*M* = 79.2, *SD* = 23.4) and week four (*M* = 73.8, *SD* = 18.1) from baseline concentrations (*M* = 57.9, *SD* = 27.3) in the kiwifruit condition. Mean vitamin C levels for the diet-as-usual condition did not vary considerably across timepoints.

Results for wellbeing (Figure 4B) showed a significant effect of time, *F*(2, 112.20) = 4.40, *p* = 0.015, and a significant overall treatment effect, *F*(1, 85.98) = 4.12, *p* = 0.05. There was a significant interaction between treatment and time, *F*(2, 112.19) = 6.16, *p* = 0.003. Mean wellbeing scores did not increase significantly from baseline (*M* = 42.1, *SD* = 5.7) at week two (*M* = 44.6, *SD* = 6.9) but were significantly increased at week four (*M* = 46.5, *SD* = 7.6) in the kiwifruit condition. Mean wellbeing scores did not change significantly across timepoints in the diet as usual condition (Table 4).

With regard to vitality, the results showed a significant time effect, *F*(2, 112.45) = 4.44, *p* = 0.014, and a significant overall interaction between treatment and time, *F*(2, 112.45) = 5.94, *p* = 0.004. However, there was not a significant overall treatment effect, *F*(1, 93.59) = 1.60, *p* = 0.21 (Figure 4C). There was a significant increase in vitality scores at week four (*M* = 26.4, *SD* = 6.3) but not at week two (*M* = 23.9, *SD* = 6.2) from baseline (*M* = 22.5, *SD* = 6.2) in the kiwifruit condition. Vitality scores did not differ significantly across timepoints in the diet-as-usual condition.

In relation to gastrointestinal symptoms (Figure 4D), the model showed a significant time effect, *F*(2, 90.60) = 4.36, *p* = 0.016, and a significant interaction between treatment and time, *F*(2, 90.60) = 6.83, *p* = 0.002. However, there was not a significant overall treatment effect, *F*(1, 22.98) = 1.0, *p* = 0.33. Mean gastrointestinal symptom ratings in the kiwifruit condition decreased significantly between baseline (*M* = 26.6, *SD* = 7.7) and week two (*M* = 22.3, *SD* = 6.4), and this was maintained at week four (*M* = 22.3, *SD* = 5.6) (Table 4). Mean symptom ratings did not vary greatly across timepoints in the diet-as-usual condition.

## 4. Discussion

This two-period, non-blinded randomised crossover trial investigated the potential for daily consumption of two SunGold kiwifruit to support psychological health through increased vitamin C intake in mood-disturbed adults. Importantly, there were no serious adverse events reported during the trial, and the intervention was well tolerated. Consuming two SunGold kiwifruit daily for 28 days was associated with a significant reduction in mood disturbance compared to usual diet. Results for the secondary outcomes of blood plasma vitamin C concentration, wellbeing, vitality and gastrointestinal symptoms all reflected that the mean change in scores was significantly greater during the kiwifruit period compared to diet-as-usual.

Results indicate that increased vitamin C concentrations from SunGold kiwifruit consumption may be a significant factor in supporting improvements in mood, wellbeing and vitality in the mood-disturbed sample. Of interest, the improvements in gastrointestinal functioning offer the possibility of an additional kiwifruit-mediated pathway towards improved psychological health. The present results are supported by previous clinical trials that found similar associations between gold kiwifruit consumption and psychological wellbeing, albeit in young adults with sub-optimal vitamin C concentrations [28,29]. In recruiting a mood-disturbed sample of 18–60-year-olds, however, the present trial extends that body of work to include a cohort that arguably stands to receive greater benefit than non-mood-disturbed populations [39]. Likewise, results for gastrointestinal functioning reflect previous findings that SunGold kiwifruit consumption was associated with reductions in gastrointestinal symptoms in healthy and clinical samples [40,41]. Acknowledging the associations between gastrointestinal functioning and mood disorders [42], the present trial augments this work by providing preliminary evidence for SunGold kiwifruit to improve mood through a reduction in gut symptom severity.

Notwithstanding the contribution of vitamin C to improved psychological health in the present trial, other nutrients in SunGold kiwifruit may have contributed to the observed improvements. SunGold kiwifruit contain relatively high concentrations of vitamin E, potassium, folate and dietary fibre, all of which are associated with improved mental health outcomes. For example, vitamin E is recognised for its anti-oxidant and anti-inflammatory actions and its important role in the modulation of depressive symptomatology [43]. Diets high in potassium are associated with greater vigour, reduced mood disturbance and improved mood [44], and dietary folate intake has been associated with lowered risk of developing depression and reduced depression severity [45,46]. Dietary fibre is associated with the positive modulation of gut microbiota, which has been implicated in the pathogenesis of depression [47]. The pathway between gut microbiota and depression is thought to be partly explained by the metabolism of tryptophan, an amino acid found in SunGold kiwifruit [48] and recognised for its role in serotonin production [49].

The association between diet quality and mental health is well established [50]. Results from observational and clinical trials have consistently demonstrated that healthy dietary patterns, such as the Mediterranean diet, characterised by a high intake of fruits, vegetables, wholegrains, legumes, seafood, nuts, seeds and olive oil, while being low in sugar, red meat, and processed and refined foods [51], are associated with a reduced risk of depression [52,53] and reduced depressive symptoms in depressed populations [39]. Dietary patterns such as the MDP have produced small yet significant reductions in depression severity in clinical populations [54]. There is almost universal consensus in the nutritional psychiatry field that further research is required to distil the constituents and mechanisms of action of dietary interventions to develop more targeted and cost-effective treatments and, importantly, to conceive low-intensity treatments that offer self-agency to individuals with mental health conditions [6,39].

The present research demonstrated the potential for a simple, single-food intervention to reduce depressive symptomatology and improve positive affective states such as vitality and wellbeing in a mood-disturbed population. Furthermore, numerous barriers exist that would challenge the capacity of many individuals to adopt and adhere to a healthy dietary pattern to support their psychological health. These include perceptions of increased cost, food literacy, extra time and effort in meal preparation and family food preferences [55]. Factors such as low self-efficacy, reduced motivation and lack of social support often experienced by depressed populations represent additional barriers to adopting a healthy diet [56]. Thus, our study provides fresh insight into the potential for a nutrient-dense fruit to positively influence psychological health through multiple pathways and, importantly, aligns with the World Health Organisation’s call for lifestyle-based interventions such as diet to constitute a first-line intervention for mental health conditions [57].

Study findings must be interpreted with consideration of the following limitations. First, although the study examined the influence of vitamin C on psychological health, vitamin C concentration was not an eligibility criterion. Despite 41.7% and 50% of participants in the kiwifruit and diet-as-usual conditions recording sub-optimal vitamin C concentrations (<50 µmol/L), respectively, at baseline, it is acknowledged that the mean vitamin C concentration of the kiwifruit and diet-as-usual conditions (*M/SD* = 57.9 µmol/L/13.5 µmol/L, and 54.4 µmol/L/20.6 µmol/L, respectively) were towards the lower end of the adequate to saturating status range (i.e., 50–75 µmol/L) [20]. Increasing vitamin C intake beyond saturation status has little or no effect on tissue concentration [58], thus creating a potential ceiling effect on mood outcomes in the present sample.

Second, other nutrients found in SunGold kiwifruit (e.g., vitamin E, potassium, folate and dietary fibre) with established associations with mental health outcomes [59,60,61] were not measured and their relative contribution remains unquantified. This does not preclude, however, that the observed improvements in mood outcomes are the direct result of vitamin C or that other nutrients in the kiwifruit acted either alone or synergistically to affect improvements. For example, vitamin C from gold kiwifruit supports the bioavailability of iron [62], a trace element associated with mood outcomes and considered essential for serotonin synthesis [63,64]. Furthermore, it is understood that vitamin E and vitamin C act synergistically to ameliorate oxidative stress, a potential mechanistic pathway in mental health disorders [47].

Third, as is common in clinical nutrition research when examining whole foods or whole-of-diet interventions, the blinding of participants and researchers is difficult, if not impossible [65]. The present study was not immune to these challenges, and participants and researchers remained aware of allocation, intervention and study methodology. Fourth, the sample in the present trial was predominantly female (~75%) and it has been reported that female participants tend to report greater psychological benefit from dietary interventions compared to all-male samples [39]. Gender-specific factors cited to explain this difference include sex differences in metabolism and body composition and socio-cultural differences in expectations regarding diet that predispose females to be more likely to adopt health behaviours compared to males [39]. Additionally, females exhibit a greater dose–response bioavailability of vitamin C than males [19]. Noted gender differences in dietary intake and behaviour [66] and in mood disorders [67] may further limit the generalisability of findings.

Finally, the mean baseline gastrointestinal symptom rating of the kiwifruit and diet-as-usual conditions (*M/SD* = 26.6 µmol/L/7.7 µmol/L, and 25.0 µmol/L/7.6 µmol/L, respectively) were in the lowest quartile (possible score range 15–95), suggesting a mild symptom profile. This may have introduced a floor effect on symptom reduction. SunGold kiwifruit-delivered dietary fibre may have conferred broader effects on gut health by increasing the diversity of dietary fibre ingested compared to a single-fibre supplement. This is supported by previous findings that noted that SunGold kiwifruit consumption resulted in fewer side effects and greater symptom reduction than a dose-matched single dietary fibre supplement [40,68]. Notwithstanding the threat of a floor effect, participants reported significant reductions in gastrointestinal symptom severity. Acknowledging the established associations between mood disturbance and gastrointestinal symptoms [69], the improvements in psychological wellbeing may have been informed by dietary fibre-induced gastrointestinal symptom reduction rather than from increased vitamin C intake.

The primary strength of this study was the decision to recruit adults with mild to moderate mood disturbance. Current clinical treatment guidelines recommend dietary interventions as one component of lifestyle-based treatment protocols for adults with sub-clinical depression [70]. Previous kiwifruit trials examining mood did not select participants based on mood status. Thus, the present work provides valuable new information on the benefits of a whole-food dietary intervention for psychological health. The use of validated measures of mood, wellbeing, vitality and gastrointestinal functioning in addition to objective measurements of vitamin C concentration at regular intervals across the trial further strengthens results. Screening for conditions that might affect vitamin C bioavailability and excluding individuals with alterations in dose or class of pharmacological agents in the lead-up to participation were additional assets. The crossover design was chosen as it served to reduce the sample size required, along with the added advantage of each participant acting as their own control. Although considered a priori as negligible, any potential carryover effect from vitamin C intake during the kiwifruit intervention was controlled for by setting the length of the washout period in line with depletion and repletion kinetics of vitamin C [71]. Recruitment and retention difficulties for studies involving mood-compromised individuals are well-documented [72]; however, the low drop-out rate and substantial interest in the study reflect positively on the study design. Highlighting the relatively benign nature and low risk of the intervention, SunGold kiwifruit was well tolerated, and no study product-related serious adverse events were reported.

The aim of the present trial was to examine the potential for SunGold kiwifruit to support psychological health in an at-risk population, namely, adults with mild to moderate mood disturbance. Targeting this population is of particular importance given that dietary interventions, and in particular single food interventions, represent a relatively low-risk, low-intensity preventative measure that can either form part of a clinician-delivered treatment programme or as a self-management approach for reducing symptoms of depression [39].

Larger randomised controlled trials with more diverse mental health, cultural, age and socio-demographic profiles are warranted. Recruiting a population-representative gender balance would support the generalisation of results and would help determine the optimum nutrient intake to prevent or reduce depressive symptoms and support the development of gender-individualised dietary interventions. For example, future SunGold research could target at-risk populations likely to benefit from increased intake of specific nutrients in SunGold kiwifruit in longitudinal studies (i.e., increased folate and vitamin C intake to reduce the incidence of perinatal depressive symptoms). An example of the potential utility of SunGold kiwifruit to support at-risk populations was demonstrated in a recent clinical trial in which participants (*n* = 20) with respiratory infection and sub-optimal vitamin C concentrations (*M* = 45 µmol/L) consumed two SunGold kiwifruit daily for six weeks [73]. Following the intervention, participants’ vitamin C concentrations were restored to adequate levels and the subset of participants (*n* = 7) with higher depression scores at baseline recorded significant decreases in depression scores (*p* = 0.03). To the extent possible, incorporating study design features that achieve full or at least partial blinding of participants and researchers would substantiate findings. Lastly, future research that identifies the individual components or micronutrient interactions that mediate the relationships between dietary intake and psychological health will contribute to the field and provide a greater understanding of the role of dietary interventions in the treatment of mental health conditions.

## 5. Conclusions

The present trial provides preliminary evidence that SunGold kiwifruit consumption improves mood, wellbeing, vitality and gastrointestinal functioning, as well as vitamin C status, in mood-compromised adults. These findings build upon the small but growing body of work uncovering the mood-enhancing properties of SunGold kiwifruit. Importantly, the present study demonstrated these effects in mood-disturbed participants, adding further weight to the potential benefits of dietary interventions, such as kiwifruit, for reducing mood symptoms. Emerging evidence suggests that while dietary interventions can realise small but significant reductions in symptoms of depression in non-clinical populations, larger effects are observed in populations with higher baseline symptoms of depression [47]. Thus, the present study makes an original contribution to the field of nutritional psychiatry by examining the effects of a whole-food nutritional intervention in a population that, based on the evidence to date, is most likely to receive benefit from dietary interventions to support psychological wellbeing. Additionally, the findings have the potential to strategically inform future policy development and treatment practices in nutritional psychiatry. Given the growing recognition of the benefits to psychological health from dietary interventions and the need to provide alternate or adjuvant options in the clinical and public policy space, the nutrient-dense SunGold kiwifruit offers an appealing, nutritious and affordable pathway to positive psychological wellbeing.

## Figures and Tables

**Table 1:** GoKiPH study eligibility criteria.

Inclusion Criteria	Exclusion Criteria
Males and females aged 18–60 years Non-smokers (within 6 months of baseline visit) Scores in the mild (20–24) to moderate (25–29) ranges on the Kessler Psychological Distress Scale at phone screening Willingness to provide written informed consent Able to access own email service Fluent in English	Allergy/intolerance to kiwifruit and/or latex Recent smoker or NRT * (within 6 months) Taking vitamin C supplements (within 3 months of baseline) Previous or current diagnosis of diabetes mellitus, bleeding disorders, iron deficiency or hypo-/hyperthyroidism Needle phobia or fainting due to fear of needles Taking prescription medication for gastrointestinal conditions (within 3 months of baseline) Initiation of, or alteration to, a course of anti-depressants, anxiolytics or antipsychotics (within 6 months of baseline) Received an investigational drug (within 3 months of baseline) Currently enrolled in any other dietary study or previously enrolled in a dietary intervention study at CSIRO involving kiwifruit

* NRT: nicotine replacement therapy.

**Table 2:** Baseline characteristics for kiwifruit and diet-as-usual conditions *.

	Kiwifruit (*n* = 24)	Diet as Usual (*n* = 25)
Female (%)	18 (75%)	19 (76%)
Age	36.8 (11.0)	36.3 (11.2)
BMI	29.1 (8.4)	29.9 (8.1)
POMS-SF TMD	27.6 (13.5)	21.7 (17.5)
Vitamin C ^	57.9 (27.3)	54.4 (20.6)
WEMWBS	42.1 (5.7)	43.8 (7.4)
SVS	22.5 (6.2)	23.5 (6.9)
GSRS	26.6 (7.7)	25.0 (7.6)

* Means (SD) unless stated. ^ µmol/L. BMI = Body Mass Index (kg/m^2^); POMS-SF TMD = Profile of Mood States—Short Form Total Mood Disturbance; WEMWBS = Warwick–Edinburgh Mental Wellbeing Scale; SVS = Subjective Vitality Scale; GSRS = Gastrointestinal Symptom Rating Scale.

**Table 3:** Linear mixed effects models for primary and secondary outcomes for sequence, treatment, time and treatment × time interaction.

Model	TMD			Plasma vC			SVS			WEMWBS			GSRS		
	β	CI	*p*	β	CI	*p*	β	CI	*p*	β	CI	*p*	β	CI	*p*
Sequence															
AB	-	-	-	-	-	-	-	-	-	-	-	-	-	-	-
BA	12	3.6, 21	0.007 *	2.3	−11, 16	0.7	−4.7	−9.1, −0.39	0.034 *	−6.3	−11, −2.0	0.006 *	1.1	−3.8, 6.0	0.6
Treatment															
Kiwifruit	-	-	-	-	-	-	-	-	-	-	-	-	-	-	-
DAU	−6.9	−13, −0.92	0.024 *	−2.7	−14, 9.1	0.7	1.4	−0.35, 3.1	0.12	1.7	−0.36, 3.8	0.10	−2.1	−5.1, 0.90	0.2
Time															
Week 2	−8.9	−15, −2.9	0.004 *	23	11, 34	<0.001 *	1.3	−0.38, 3.0	0.13	2.5	0.46, 4.5	0.017 *	−4.3	−6.6, −1.9	<0.001 *
Week 4	−18	−24, −12	<0.001 *	17	6.0, 29	0.003 *	3.9	2.2, 5.7	<0.001 *	4.2	2.2, 6.3	<0.001 *	−4.6	−7.0, −2.3	<0.001 *
Treatment × Time															
DAU × Week 2	12	3.4, 20	0.006 *	−19	−35, −3.0	0.02 *	−1.8	−4.3, 0.58	0.13	−4.5	−7.4, −1.6	0.003 *	3.7	0.36, 7.0	0.03 *
DAU × Week 4	19	10, 27	<0.001 *	−20	−36, −4.0	0.015 *	−4.3	−6.7, −1.8	<0.001 *	−4.4	−7.3, −1.5	0.003 *	6.2	2.8, 9.5	<0.001 *

TMD = Total Mood Disturbance; vC = vitamin C; SVS = Subjective Vitality Scale; WEMWBS = Warwick-Edinburgh Mental Wellbeing Scale; GSRS = Gastrointestinal Symptom Rating Scale; CI = Confidence interval; DAU = Diet As Usual. * significant at *p* = 0.05.

**Table 4:** Means and standard deviations for outcome measures at baseline, week 2 and week 4 for kiwifruit and diet-as-usual conditions.

	Kiwifruit			Diet As Usual		
	Baseline (*n* = 24)	Week 2 (*n* = 24)	Week 4 (*n* = 23)	Baseline (*n* = 25)	Week 2 (*n* = 24)	Week, (*n* = 23)
Vitamin C *						
Mean (SD)	57.9 (27.29)	79.2 (23.42)	73.8 (18.06)	54.4 (20.60)	56.5 (26.47)	49.7 (27.34)
POMS-SF TMD						
Mean (SD)	27.6 (13.46)	18.6 (13.53)	9.6 (11.96)	21.7 (17.49)	24.2 (17.14)	21.3 (16.52)
WEMWBS						
Mean (SD)	42.1 (5.71)	44.6 (6.93)	46.5 (7.58)	43.8 (7.40)	41.9 (7.35)	43.8 (7.93)
SVS						
Mean (SD)	22.5 (6.15)	23.9 (6.16)	26.4 (6.30)	23.5 (6.86)	22.9 (6.83)	23.5 (5.95)
GSRS						
Mean (SD)	26.6 (7.73)	22.3 (6.43)	22.3 (5.55)	25.0 (7.56)	24.3 (6.73)	26.1 (9.57)

* µmol/L; POMS-SF TMD = Profile of Mood States-Short Form Total Mood Disturbance; WEMWBS = Warwick–Edinburgh Mental Wellbeing Scale; SVS = Subjective Vitality Scale; GSRS = Gastrointestinal Symptom Rating Scale.

## Data Availability

The raw data supporting the conclusions of this article will be made available by the authors on request due to legal obligations to obtain prior consent to release.

## Abbreviations

The following abbreviations are used in this manuscript:

BMI	Body Mass Index
CHMREC	CSIRO Health and Medical Research Ethics Committee
CSIRO	Commonwealth Scientific and Industrial Research Organisation
DAU	Diet-As-Usual
GoKiPH	Gold Kiwifruit and Psychological Health
GSRS	Gastrointestinal Symptom Rating Scale
NHMRC	National Health and Medical Research Council
NRT	Nicotine Replacement Therapy
NZIFPR	New Zealand Institute for Food and Plant Research
POMS-SF	Profile of Mood States—Short Form
TMD	Total Mood Disturbance
WEMWBS	Warwick–Edinburgh Mental Wellbeing Scale
